# Protein structure search and local structure characterization

**DOI:** 10.1186/1471-2105-9-349

**Published:** 2008-08-22

**Authors:** Shih-Yen Ku, Yuh-Jyh Hu

**Affiliations:** 1 Department of Computer Science, National Chiao Tung University, 1001 University Rd. Hsinchu, Taiwan; 2 Institute of Statistics, Academia Sinica, Taipei, Taiwan; 3 Institute of Biomedical Engineering, National Chiao Tung University, 1001 University Rd. Hsinchu, Taiwan

## Abstract

**Background:**

Structural similarities among proteins can provide valuable insight into their functional mechanisms and relationships. As the number of available three-dimensional (3D) protein structures increases, a greater variety of studies can be conducted with increasing efficiency, among which is the design of protein structural alphabets. Structural alphabets allow us to characterize local structures of proteins and describe the global folding structure of a protein using a one-dimensional (1D) sequence. Thus, 1D sequences can be used to identify structural similarities among proteins using standard sequence alignment tools such as BLAST or FASTA.

**Results:**

We used self-organizing maps in combination with a minimum spanning tree algorithm to determine the optimum size of a structural alphabet and applied the k-means algorithm to group protein fragnts into clusters. The centroids of these clusters defined the structural alphabet. We also developed a flexible matrix training system to build a substitution matrix (TRISUM-169) for our alphabet. Based on FASTA and using TRISUM-169 as the substitution matrix, we developed the SA-FAST alignment tool. We compared the performance of SA-FAST with that of various search tools in database-scale search tasks and found that SA-FAST was highly competitive in all tests conducted. Further, we evaluated the performance of our structural alphabet in recognizing specific structural domains of EGF and EGF-like proteins. Our method successfully recovered more EGF sub-domains using our structural alphabet than when using other structural alphabets. SA-FAST can be found at .

**Conclusion:**

The goal of this project was two-fold. First, we wanted to introduce a modular design pipeline to those who have been working with structural alphabets. Secondly, we wanted to open the door to researchers who have done substantial work in biological sequences but have yet to enter the field of protein structure research. Our experiments showed that by transforming the structural representations from 3D to 1D, several 1D-based tools can be applied to structural analysis, including similarity searches and structural motif finding.

## Background

Genome sequencing projects continue to produce amino acid sequences; however, understanding the biological roles played by these putative proteins requires knowledge of their structure and function [1]. Despite that empirical structure determination methods have provided structural information for some proteins, computational methods are still required for the large number of proteins whose structures are difficult to determine experimentally. And while the primary sequence should contain the folding guide for a given protein, our ability to predict the three-dimensional (3D) structure from the primary sequence alone remains limited. Some *ab initio *methods do not require such information, but the application of these methods is often limited to small proteins [2,3].

Structure alignment research has led to the discovery of homologues of novel protein structures. And, although many structure alignment tools have been developed, such as CE [4], DALI [5], VAST [6], MAMMOTH [7], FATCAT [8], and Vorolign [9], we wanted to provide a different perspective on protein structure analysis. Previous studies of protein structures have shown the importance of repetitive secondary structures, particularly *α*-helices and *β*-sheets, in overall structure determination. Together with variable coils, these structures constitute a basic three-letter structural alphabet that has been used in the development of early-generation secondary structure prediction algorithms (such as GOR [10]) as well as more recent-generation algorithms. These newer algorithms have been applied to neural networks, homology sequences, and discriminative models [11-14], and their accuracy in predicting secondary structure approaches 80%. However, despite this predictive accuracy, the three-letter alphabet does not contain the information necessary to approximate more refined 3D reconstructions.

The recent rapid increase in the number of available protein structures has allowed more precise and thorough studies of protein structures. Several authors have developed more complex structural alphabets that incorporate information about the heterogeneity of backbone protein structures by using subsets of small protein fragments that are observed frequently in different protein structure databases [15-17]. The alphabet size varies from several letters to about 100 letters [18]. For example, Unger *et al*. [19] and Schuchhardt *et al*. [20] used k-means methods and self-organizing maps (SOMs), respectively, to identify the most common folds, but the number of clusters generated was too large to have substantial predictive value. By applying autoassociative neural networks, Fetrow *et al*. defined six clusters that represent super-secondary structures which subsume the classic secondary structures [21]. Bystroff and Baker produced similar short folds of different lengths and grouped them into 13 clusters that they used to predict 3D structure [22]. Camproux *et al*. developed a hidden Markov model (HMM) approach that accounted for the Markovian dependence to learn the geometry of the structural alphabet letters and the local rules for the assembly process [23]. Fixing the alphabet size to 23 letters, Yang & Tung applied a nearest-neighbor algorithm on a (*κ*, *α*)-map of structural segments to identify the 23 groups of segments used in their alphabet [24]. More details about these local structures can be found in a recent review [25].

In this study, we developed a flexible pipeline for protein structural alphabet design based on a combinatorial, multi-strategy approach. Instead of applying cross-validation [22] or Markovian processes [15] to refine the clusters directly, we used SOMs and Bayesian Information Criterion (BIC) to determine the optimum size of structural alphabet. We then applied the k-means algorithm [26] to group protein fragments into clusters, forming the bases of our structural alphabet. Moreover, unlike most other works that built substitution matrices for alphabets based on known blocks of aligned proteins, we used a matrix training framework that generated matrices automatically without depending on known alignments. An expressive structural alphabet allows us to quantify the similarities among proteins encoded in the appropriate letters. It also enables the primary representation of 3D structures using standard 1D amino acid sequence alignment methods. To demonstrate the feasibility of our new method, we verified the application of the alphabet produced by our pipeline and the trained substitution matrix to a widely used 1D alignment tool, FASTA [27]. We conducted several experiments using the same datasets used in other recently published works and evaluated the performance of our tool in database-scale searches. In addition to investigating whether our alphabet and matrix worked well with 1D alignment tools in database searches, we evaluated the ability of our structural alphabet to characterize local structural features.

## Results

### Structural alphabet

By combining SOMs, minimum spanning trees, and k-means clustering, we developed a multi-strategy approach to designing a protein structural alphabet. To derive an appropriate substitution matrix for the new alphabet, we developed a matrix training framework that would automatically refine an initial matrix repeatedly until it converged. Unlike some previous works that presumed the size of the alphabet [23], our method determined the alphabet size autonomously and statistically. Various experiments were conducted to evaluate our methodology.

The SOM is an unsupervised inductive learner and can be viewed as topology preserving mapping from input space onto the 2D grid of map units [28]. The number of map units in SOMs defines an inductive bias [29], as does the number of hidden units for the feedforward artificial neural networks, and it affects the clustering results. By systematically varying the number of SOM map units and applying BIC, we identified the most frequent number of clusters that maximized the BIC and used this number to define the size of the alphabet. We tested SOMs ranging in size from 10 × 10 to 200 × 200, ultimately defining the size of our alphabet at 18 letters. The relationship between number of clusters found and number of SOM map units used is summarized in Table 1.

**Table 1:** Relationship between the number of clusters found and the number of SOM map units used

SOM map size	Number of clusters	SOM map size	Number of clusters
10 × 10	6	110 × 110	24
20 × 20	9	120 × 120	19
30 × 30	10	130 × 130	21
40 × 40	12	140 × 140	22
50 × 50	15	150 × 150	18
60 × 60	13	160 × 160	15
70 × 70	14	170 × 170	21
80 × 80	18	180 × 180	18
90 × 90	18	190 × 190	18
100 × 100	20	200 × 200	18

Our analysis determined that among the number of clusters that maximized the *BIC*, 18 clusters occurred most frequently. Thus, we assigned 18 letters to our alphabet.

**Figure 1:**
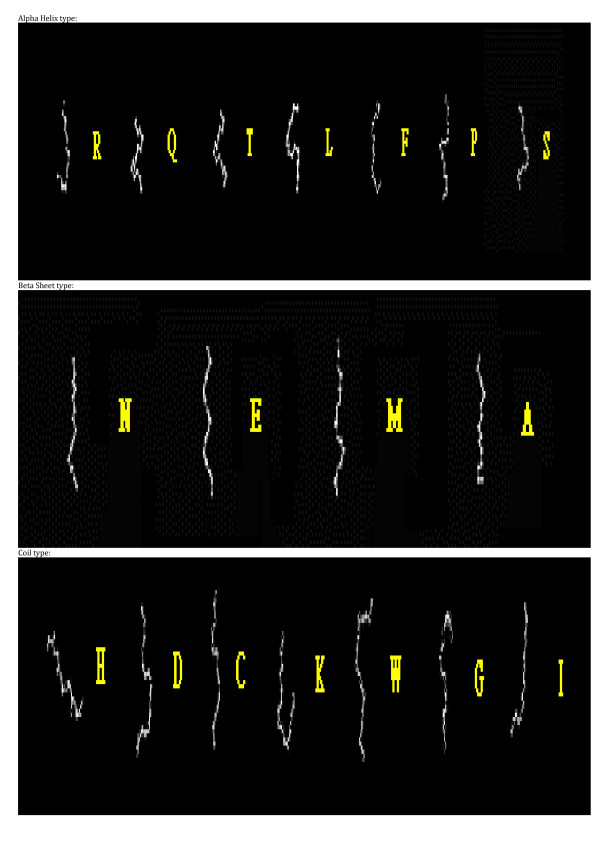
The 3D conformation of the representative segment for each alphabet letter.

**Figure 2:**
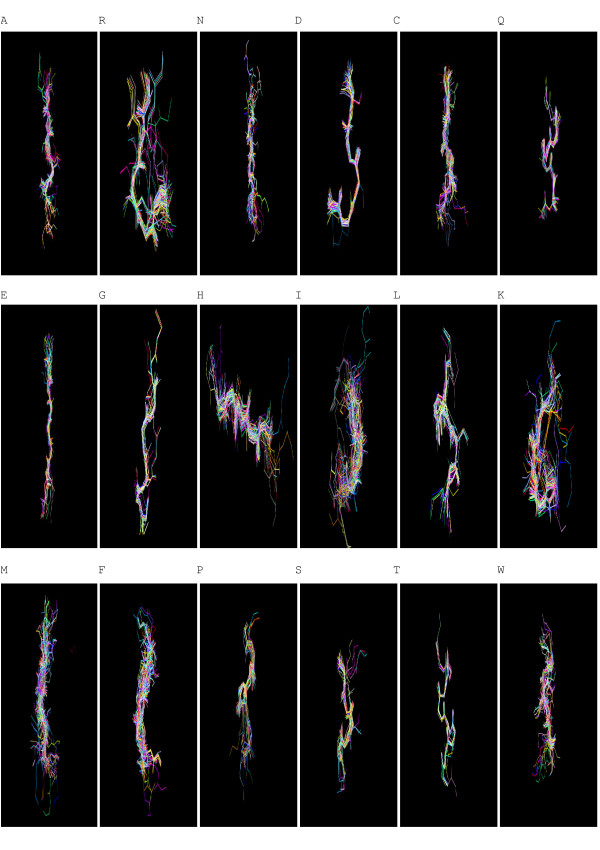
Superimposition of protein segments in the 18 clusters.

To verify whether fragments were assigned to the same cluster by the various SOMs, we analyzed those SOMs (with varying numbers of map units) that produced 18 clusters, including SOMs sized 80 × 80, 90 × 90, 190 × 190 units, etc. We calculated the overlap level between any two of the SOMs, defined as percentage of fragments that belonged to the same cluster. The average overlap between all pairs of SOMs for each of the 18 clusters was over 90%, indicating that these clusters were very consistent (Table 2). Table 3 and 4 display the within-cluster Euclidean distance, defined as the average distance of each segment to the center, and the center-to-center Euclidean distance for the 18 protein fragment clusters found by our method and by SOM alone, respectively. The average Phi/Psi angles (i.e. the Phi/Psi angles of the centroid) for the 18 clusters are presented in Table 5. As indicated in Table 3 and 4, the within-cluster Euclidean distances for our clusters were smaller than those of the SOM clusters, which suggested that our 18 clusters were more coherent. On the other hand, the center-to-center distances for our clusters were larger than those of the SOM clusters, indicating that our clusters were better separated from each other. The 3D conformation of the representative segment for each alphabet letter is illustrated in Figure 1 and the superimposition of protein segments is shown in Figure 2. To verify that these representative segments could be the building blocks for protein structures, we analyzed the frequency of their occurrence in four major structural classes according to the Structural Classification of Proteins (SCOP): all-alpha, all-beta, alpha/beta, and alpha+beta [30]. The frequency of each category of segments is presented in Table 6. The alpha helix segments represented by alphabet letters T, P, and R occurred more often in the all-alpha class than did the other segments. Similarly, more beta sheet segments, such as N, E, and A, were found in the all-beta class. In both the alpha/beta and alpha+beta classes, most of the segments were found to be either alpha helices or beta sheets.

**Table 2:** The average overlap between all pairs of SOMs that produced 18 clusters of fragments

**Cluster**	1	2	3	4	5	6	7	8	9	10	11	12	13	14	15	16	17	18
**Overlap**	99.8	98.4	96.7	97.4	97.4	94.3	99.1	95.0	97.8	94.6	99.8	95.6	96.7	95.3	95.7	98.2	96.3	95.5

**Table 3:** Summary of the within-cluster Euclidean distance and the center-to-center Euclidean distance for 18 protein fragment clusters found by our alphabet design pipeline

	**Within-Cluster**		**Center-to-Center**																	
	**Mean**	**SD**	18	17	16	15	14	13	12	11	10	9	8	7	6	5	4	3	2	1
1	116.6	37.2	252.3	300.4	330.1	242.8	181.7	182.1	317.6	327.7	415.4	266.3	329.0	181.7	242.5	262.2	273.6	253.4	193.2	0
2	238.7	38.5	315.8	226.6	272.7	197.4	243.3	227.2	285.3	270.5	346.1	283.9	285.4	261.3	189.5	182.3	215.0	296.0	0	
3	264.7	29.8	219.7	279.8	193.6	220.6	190.4	284.1	251.1	292.9	413.2	195.1	237.6	181.4	324.4	234.1	285.9	0		
4	319.3	41.5	297.8	297.0	270.7	285.5	311.5	288.6	286.9	317.1	352.2	302.9	184.3	250.7	256.2	193.3	0			
5	250.4	39.7	248.6	268.9	190.2	238.1	302.2	280.1	258.5	287.2	406.6	267.2	258.8	192.8	229.0	0				
6	257.5	28.0	220.4	174.2	242.3	180.4	262.8	266.2	264.4	229.1	310.3	322.3	270.9	308.6	0					
7	72.2	20.4	220.8	356.7	289.2	297.5	266.1	244.8	307.2	361.1	478.3	248.9	316.8	0						
8	282.2	31.0	275.3	214.2	186.1	218.9	259.1	335.6	258.2	253.8	286.9	273.9	0							
9	320.9	27.9	275.8	287.6	250.7	244.5	222.6	292.2	286.7	307.3	354.3	0								
10	148.8	26.1	406.3	243.1	334.4	286.3	333.5	361.8	293.2	240.8	0									
11	97.1	43.4	290.4	169.5	214.8	178.9	248.7	238.3	270.4	0										
12	272.0	32.7	259.7	226.6	200.7	218.7	269.1	325.6	0											
13	133.6	33.2	291.2	309.3	334.3	267.6	230.5	0												
14	272.8	31.4	255.5	206.2	258.7	145.3	0													
15	106.2	32.3	241.1	76.8	162.1	0														
16	109.0	39.1	221.8	172.6	0															
17	33.2	23.2	272.9	0																
18	146.2	38.2	0																	

**Table 4:** Summary of the within-cluster Euclidean distance and the center-to-center Euclidean distance for 18 protein fragment clusters found by the SOM alone

	**Within-Cluster**		**Center-to-Center**																	
	**Mean**	**SD**	18	17	16	15	14	13	12	11	10	9	8	7	6	5	4	3	2	1
1	129.1	38.9	220.9	270.9	302.4	202.7	175.7	161.9	277.2	295.9	381.4	233.8	307.6	181.6	234.8	223.0	263.6	250.5	164.4	0
2	242.7	39.4	304.8	198.1	265.0	192.0	201.2	217.5	241.3	237.1	309.9	244.0	247.9	259.6	165.5	169.5	189.0	273.5	0	
3	265.8	29.8	180.3	276.0	168.3	177.9	169.5	266.4	237.3	256.2	397.2	185.0	218.6	156.4	298.5	224.5	280.4	0		
4	327.1	41.4	261.7	275.8	265.8	241.5	298.8	273.8	250.9	297.5	321.2	266.9	182.9	215.2	250.4	156.8	0			
5	251.9	39.6	206.8	223.7	150.2	207.1	300.9	258.4	217.8	274.8	400.7	227.2	243.6	167.1	227.6	0				
6	260.7	29.2	202.0	158.5	235.8	137.3	248.4	225.3	258.8	205.8	304.7	300.23	235.0	297.0	0					
7	75.7	20.5	191.9	323.9	243.6	280.5	238.4	199.0	292.7	346.6	463.2	247.1	291.3	0						
8	291.4	30.8	250.4	196.2	144.8	203.1	245.8	322.9	245.4	226.8	265.6	272.7	0							
9	329.1	27.9	275.3	251.6	219.3	200.6	197.1	263.8	278.1	272.5	342.3	0								
10	157.9	27.4	364.8	240.4	310.3	262.0	292.9	329.8	266.4	234.3	0									
11	113.8	45.8	244.9	156.7	190.2	167.5	224.8	213.6	254.9	0										
12	283.0	32.4	215.7	205.4	197.6	191.5	239.0	299.2	0											
13	170.3	29.5	277.6	272.6	322.4	252.2	188.8	0												
14	277.8	32.6	238.5	179.7	239.8	99.5	0													
15	111.2	33.1	210.6	59.5	161.6	0														
16	114.05	38.4	219.4	146.8	0															
17	36.2	24.8	228.6	0																
18	158.5	37.4	0																	

**Table 5:** The average Phi/Psi angles (i.e. the Phi/Psi angles of the centroid) for the 18 clusters found by our alphabet design pipeline

	**Φ(i)**	**Φ(i+1)**	**Φ(i-1)**	**Φ(i+2)**	**ψ(i)**	**ψ(i-1)**	**ψ(i-2)**	**ψ(i+1)**
**1(A)**	-97.99	-70.43	-104.52	-79.77	132.99	118.98	132.37	-44.26
**2(R)**	-67.81	-67.48	-92.52	-69.17	-52.78	134.75	96.12	-35.69
**3(N)**	-98.66	-99.17	-83.46	-104.16	132.56	75.64	-36.97	134.01
**4(D)**	90.39	-63.35	-93.54	-84.31	-5.43	97.71	115.22	94.64
**5(C)**	-88.09	-102.50	-93.58	-97.49	-51.56	88.66	106.12	133.27
**6(Q)**	-65.87	-69.19	-85.50	-59.89	-35.12	-50.41	129.98	-37.57
**7(E)**	-107.28	-96.08	-107.66	-105.96	132.71	130.92	133.88	133.06
**8(G)**	89.16	-93.43	-62.92	-90.25	20.65	0.22	-32.50	85.94
**9(H)**	-91.05	-90.16	91.91	-91.53	100.48	103.36	5.40	75.56
**10(I)**	58.59	56.79	55.50	54.75	-42.38	-38.76	-47.77	-48.46
**11(L)**	-71.08	-84.21	-65.92	87.57	-21.11	-29.95	-31.80	20.00
**12(K)**	-83.07	95.78	-69.02	-91.34	9.50	-9.18	-5.50	100.52
**13(M)**	-88.72	-64.82	-95.72	91.27	100.65	113.69	107.43	0.70
**14(F)**	-87.36	-71.63	-75.80	-68.31	134.69	58.97	-35.87	-49.72
**15(P)**	-96.95	-78.84	-75.71	-78.03	4.07	2.17	-33.25	-25.92
**16(S)**	-83.07	-95.71	-63.62	-97.87	-28.27	-28.59	-38.35	126.57
**17(T)**	-63.55	-65.43	-62.97	-68.03	-42.53	-41.88	-42.16	-38.34
**18(W)**	-105.06	-91.96	-78.47	-94.14	122.89	-83.40	109.64	99.64

**Table 6:** Frequency of occurrence of the protein segments defined by our alphabet in four main SCOP classes

	All alpha		All beta		alpha/beta		alpha+beta	
Letter	Count	Percentage	Count	Percentage	Count	Percentage	Count	Percentage
A	54859	2.95	255473	8.83	278238	5.46	161041	6.07
R	91363	4.92	148361	5.13	270345	5.31	145619	5.49
N	76176	4.10	309834	10.71	340682	6.69	202555	7.64
D	21055	1.13	127159	4.39	112078	2.20	66959	2.53
C	34856	1.88	172334	5.96	193952	3.81	102632	3.87
Q	102444	5.51	111333	3.85	271893	5.34	138081	5.21
E	58672	3.16	782607	27.06	620717	12.18	427778	16.14
G	42350	2.28	72105	2.49	147390	2.89	76968	2.90
H	39017	2.10	115542	3.99	163319	3.21	89203	3.37
I	3547	0.19	6607	0.23	9449	0.19	5739	0.22
L	49312	2.65	40909	1.41	141605	2.78	65856	2.48
K	43582	2.35	58687	2.04	146869	2.88	70549	2.66
M	16727	0.90	127070	4.39	110318	2.17	67912	2.56
F	70718	3.81	89366	3.09	179145	3.52	91702	3.46
P	104364	5.62	54939	1.91	192654	3.78	87149	3.29
S	76080	4.10	83725	2.89	173935	3.41	91160	3.44
T	937938	50.49	149259	5.17	1551585	30.46	651525	24.58
W	34533	1.86	186476	6.46	190001	3.72	108460	4.09
**Total**	**1857593**	**100.00**	**2891786**	**100.00**	**5094175**	**100.00**	**2650888**	**100.00**

### TRISUM – Substitution matrix

**Figure 3:**
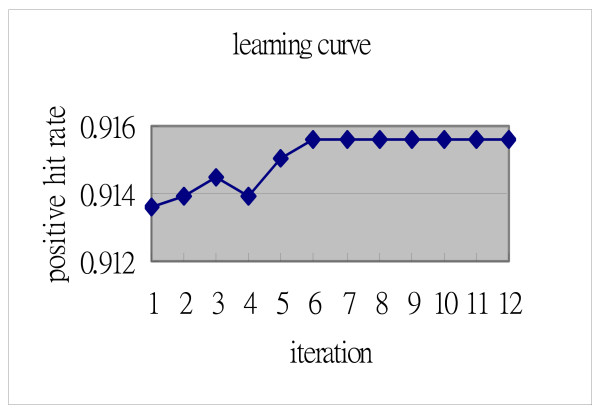
**Example learning curve of matrix training**. The average positive hit rate converged at 0.9153 with the learning rate set to 0.5.

**Figure 4:**
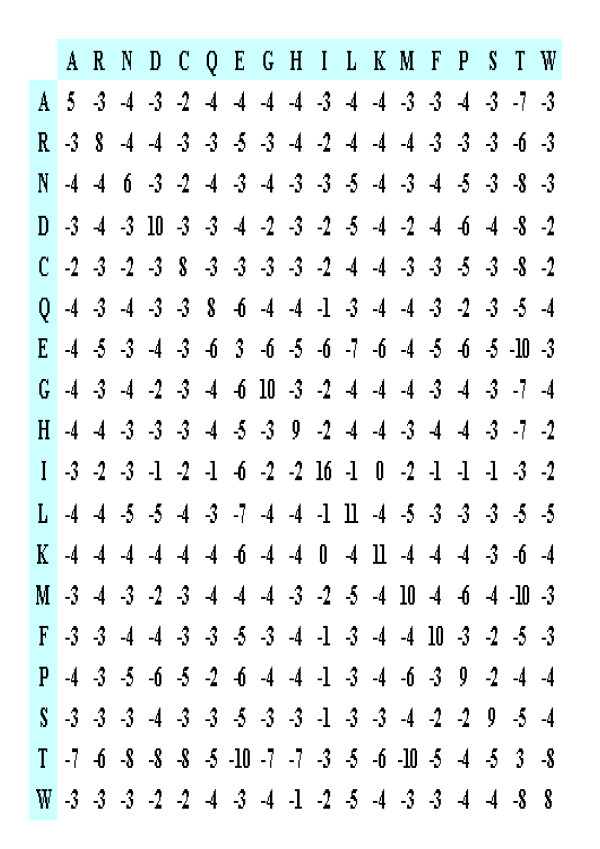
The substitution matrix TRISUM-169.

Most approaches to constructing substitution matrices require the alignment of known proteins [24,31,32]. Because alignments are not always available and their validity can be dubious, we used a self-training strategy to build the substitution matrix for our new structural alphabet. This training framework had a flexible and modular design, and unlike most other approaches, it did not rely on the pre-alignment of protein sequences or structures. Different training data or alignment tools can be incorporated into this framework to generate appropriate matrices under various circumstances. In this study, we used the non-redundant proteins contained in SCOP1.69 with sequence similarity of less than 40% for training, excluding those proteins in SCOP-894 and the 50 test proteins (see details below) to ensure that the training data and the testing data did not overlap. We defined the positive hit rate of a query as the ratio of the number of positive hits to the size of the family the query belonged to. As we iterated each training protein (as a query), we refined the matrix until we could no longer increase the average positive hit rate of all the proteins. We tried different learning rates ranging from 0.25 to 1.00. The final average positive hit rates under different learning rates were similar, ranging between 0.9112 and 0.9153. An example of the learning curve of matrix training is presented in Figure 3. We selected the converged matrix with the maximum positive hit rate with the learning rate set to 0.50. We named this matrix TRISUM-169 (TRained Iteratively for SUbstitution Matrix-SCOP1.69), as shown in Figure 4.

### Comparison with other tools

Several protein structure search tools based on 1D alignment algorithms have been developed, including SA-Search [33], YAKUSA [34], and 3D-BLAST [24]. Yang and Tung tested 3D-BLAST on the SCOP database scan task [24]. They prepared a protein query dataset named SCOP-894 from SCOP 1.67 and 1.69; this dataset contains 894 proteins with <95% sequence similarity. We tested SA-FAST on the same dataset in order to allow direct comparison (Table 7). The results indicated that SA-FAST outperformed 3D-BLAST and PSI-BLAST in the test of the SCOP-894 query dataset.

**Table 7:** SA-FAST versus 3D-BLAST and PSI-BLAST in SCOP structural function assignment accuracy for the SCOP-894 protein dataset

Class	894 proteins	Accuracy^a ^(894 proteins)			Accuracy (sequence identity <25%)		
		
	Number of queries	SA-FAST	3D-BLAST	PSI-BLAST	SA-FAST	3D-BLAST	PSI-BLAST
All alpha	161	99.27	94.41	94.41	95.83	75.00	66.67
All beta	199	95.12	94.47	93.97	87.32	77.55	73.33
*α*/*β*	292	97.58	97.26	91.44	95.68	87.88	65.77
*α*+*β*	242	95.13	94.63	88.84	93.81	83.33	60.87

^a^The top-ranked family in the hit list of a query was used as the predicted family. Accuracy is the percentage of times that the family was correctly predicted.

We also used the same 50 proteins selected from SCOP95-1.69 that were used by Yang & Tung to compare SA-FAST with 3D-BLAST, PSI-BLAST, YAKUSA, MAMMOTH, and CE, in search time, predictive accuracy, and precision. Other search tools exist, such as PBE [35], SA-Search [33], and Vorolign [9], but because they either could not be tested on the SCOP database directly or the versions of their databases provided were too old (e.g. ASTRAL in PBE derived from SCOP-1.65, Vorolign server only scans SCOP40-1.69), these tools were not used in the comparisons. The results showed that SA-FAST outperformed the other two BLAST-based search tools (i.e. 3D-BLAST and PSI-BLAST) and another structure search tool that describes structures as 1D sequences (YAKUSA) in both predictive accuracy and precision (Table 8). Additionally, SA-FAST was comparably accurate and precise as the structural alignment tools MAMMOTH and CE. Regarding search time (using one Intel Pentium 2.8 GHz processor and 512 Mbytes of memory), Table 8 clearly indicates that SA-FAST was far more efficient than were the structural alignment tools MAMMOTH and CE.

**Table 8:** Comparison between SA-FAST, 3D-BLAST, PSI-BLAST, YAKUSA, MAMMOTH, and CE on 50 proteins selected from SCOP95-1.69

Search tool	Average time required for a query (sec)	Relative to SA-FAST	Accuracy^a ^(%)	Average precision^b ^(%)
SA-FAST	1.15	1.00	96	90.80
3D-BLAST	1.30	1.13	94	85.20
PSI-BLAST	0.48	0.42	84	68.16
YAKUSA	8.88	7.72	90	74.86
MAMMOTH	1834.18	1594.94	100	94.01
CE	22053.32	19176.80	98	90.78

^a^The top-ranked family in the hit list of a query was used as the predicted family. Accuracy is the percentage of times that the family was correctly predicted.

^b^The precision is defined as T/H, where T is the number of true hit structures in the hit list, and H is the total number of structures in the hit list.

**Figure 5:**
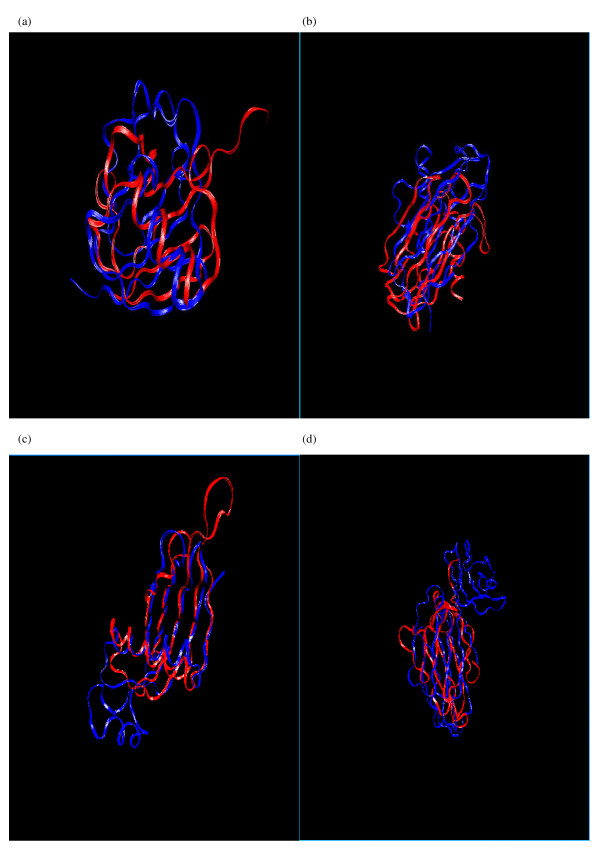
**Superimposition examples based on alignments identified by SA-FAST**. (a) 1fxiA & 1ubq_ (b) 2azaA & 1paz_ (c) 1cewI & 1molA (d) 1cid_ & 2rhe.

To further evaluate the predictive validity of our alphabet, we examined pairwise alignment of difficult cases based on the number of residues aligned and the superposition root mean square deviation (RMSD). To avoid alignment process bias and to maintain consistency in our analysis of various structural alphabets, we applied the same FASTA-based alignment algorithm [27] in the alignment tests. We tested the alphabets and substitution matrices used in PBE-align, 3D-BLAST, and SA-FAST on ten difficult cases of previously studied pairwise alignments and compared the results with those produced using VAST, DALI, CE, and FATCAT [8,36]. Based on the alignments obtained using different alphabets and matrices, we used VMD [37] to calculate the superposition RMSD for PBE-align, 3D-BLAST, and SA-FAST. Table 9 shows that our alphabet had the lowest average RMSD per aligned residue among the three structural alphabets in the ten difficult alignment tests. Figure 5 shows four superimposition examples based on our structural alphabet.

**Table 9:** Results of ten difficult cases of pairwise alignment

**Protein 1**	**Protein 2**	**VAST**	**DALI**	**CE**	**FATCAT**	**Yang & Tung's**	**de Brevern et al.'s**	**Our SA**
1fxia	1ubq	48(2.10)	60(2.60)	64(3.80)	63(3.01)	59(2.76)	76(2.89)	58(2.64)
1ten	3hhrb	78(1.60)	86(1.90)	87(1.90)	87(1.90)	57(2.57)	73(2.31)	90(2.24)
3hlab	2rhe_	-	63(2.50)	85(3.50)	79(2.81)	54(2.65)	78(3.01)	79(2.87)
2azaa	1paz_	74(2.20)	81(2.50)	85(2.90)	87(3.01)	70(2.34)	57(2.23)	87(2.40)
1cewi	1mola	71(1.9)	81(2.30)	69(1.90)	83(2.44)	52(2.37)	53(2.35)	61(1.83)
1cid_	2rhe_	85(2.20)	95(3.30)	94(2.70)	100(3.11)	54(2.75)	53(2.49)	55(2.08)
1crl_	1ede	-	211(3.40)	187(3.20)	269(3.55)	167(3.35)	120(3.47)	187(3.25)
2sim_	1nsba	284(3.80)	286(3.80)	264(3.00)	286(3.07)	121(2.75)	121(2.96)	137(3.2)
1bgea	2gmfa	74(2.50)	98(3.50)	94(4.10)	100(3.19)	27(3.34)	77(2.8)	78(2.72)
1tie_	4fgf_	82(1.70)	108(2.00)	116(2.90)	117(3.05)	91(3.15)	62(3.45)	115(3.05)
**Average RMSD/aligned-residues**		**0.0226**	**0.0238**	**0.0261**	**0.0229**	**0.0373**	**0.0363**	**0.0278**

The number of residues aligned and the RMSD (in parentheses) are shown. The last row displays the average RMSD per aligned residue. Except for PBE-align, 3D-BLAST, and SA-FAST, the results of the methods were adopted from [36].

**Figure 6:**
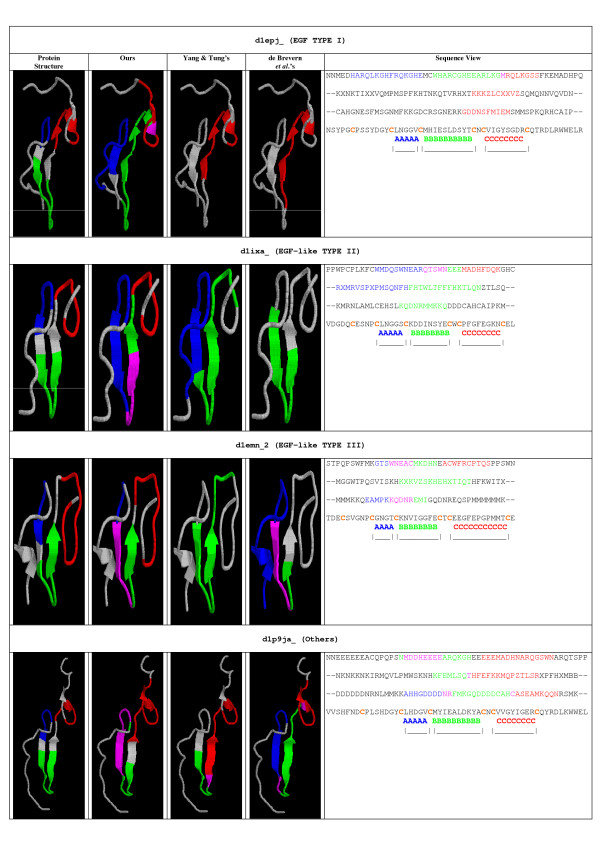
**Examples of structural motifs corresponding to EGF sub-domains**. We colored the sub-domains A, B, and C in blue, green, and red, respectively. The motifs that corresponded to EGF sub-domains, using our structural alphabet and those of Yang & Tung and de Brevern *et al*., were also highlighted in blue, green, and red. The overlapping region between motifs was colored purple. In the sequence view, the first three sequences are EGF protein represented by our structural alphabet, the alphabet of Yang & Tung, and the alphabet of de Brevern *et al*., respectively. The fourth is the amino acid sequence with the cysteines highlighted in orange. The sub-domains are marked at the bottom.

Local structure conservation in putative active sites can reflect biological meaning and these types of structural patterns can be used to predict protein function [19], e.g., the binding sites for metal-binding proteins [38]. Conserved local structural features can be identified in various ways and described using different representations. Because of the aforementioned advantages to 1D representation, we wanted to evaluate the feasibility of describing structural domains/sub-domains using our structural alphabet. Because there is no motif finding tool specifically designed for protein structural alphabets, we applied the motif finding programs available to evaluate the feasibility of using structural alphabets to characterize local structure features. Currently, we use the motif finding program, MEME [39] to identify common structural motifs in protein families. We tested our method on a well-known protein family, the epidermal growth factor (EGF)/EGF-like family. Based on the information published in literature or recorded in databases, we could verify whether the protein domains/sub-domains in EGF/EGF-like proteins could be described accurately using structural alphabets. EGF domains comprise extracellular protein modules described by 30–40 amino acids primarily stabilized by three disulfide bonds. Homologies and functional data suggest that these domains share some common functional features. If we number the cysteine residues as Cys1 to Cys6, where Cys1 is the closest to the N-terminus, the regularity of cysteine spacing defines three regions: A, B, and C. Based on the conservation in sequence and length of these regions, the homologies have been classified into three different categories [40]. We first described the 227 proteins in the EGF-type module family of SCOP 1.69 using our alphabet and the alphabets of Yang & Tung's [24] and de Brevern et al. [16,35]. We then used MEME to identify the common motifs corresponding to the A, B, and C sub-domains. According to InterPro [41], 24 of these proteins were exclusively of *EGF Type-1*, 74 were of *EGF-like Type-2*, and 117 belonged to *EGF-like Type-3 *only. We classified the remaining 12 proteins as *Others*. Sub-domain A was typically composed of five to six residues in Types 1 and 2, sub-domain B usually contained 10–11 residues in Type-1 but was consistently three residues shorter than in Type-2. Sub-domain C was conserved in length and contained four or five specific residues in Type-1 and Type-2 [40]. The sub-domains in *EGF-like Type-3 *were less conserved. A found motif was considered to correspond to a sub-domain if more than one-half of the residues in the sub-domain were included in the motif. If any single motif correctly corresponded to a sub-domain, we claimed that this sub-domain was recovered successfully (that is, a hit). The results of the motifs found are summarized in Table 10 and 11. They show that MEME was able to identify more EGF sub-domains using our structural alphabet than using the alphabets of Yang & Tung or de Brevern *et al*. One example of each EGF group is shown in Figure 6, including the structures with highlighted sub-domains. Using our alphabet, MEME identified meaningful motifs that covered all three sub-domains in the EGF examples (Figure 6); however, using Yang & Tung's or de Brevern *et al*.'s alphabets, the motifs found covered only one or two sub-domains.

**Table 10:** Comparison between our structural alphabet (used in SA-FAST) and those of Yang & Tung (used in 3D-BLAST) and de Brevern *et al*. (converted by PBE-T, a facility associated with PBE-align) for describing motifs found by MEME within the EGF family

		**Our SA**						**Yang & Tung's**						**de Brevern *et al*.'s**					
**Sub-domain Type**		**A**		**B**		**C**		**A**		**B**		**C**		**A**		**B**		**C**	

**EGF proteins**	**No**.^**a**^	**Hits**^**b**^	**Cov**^**c**^	**Hits**	**Cov**	**Hits**	**Cov**	**Hits**	**Cov**	**Hits**	**Cov**	**Hits**	**Cov**	**Hits**	**Cov**	**Hits**	**Cov**	**Hits**	**Cov**

**Type 1**	24	23	95.8	22	91.7	23	95.8	11	45.8	21	87.5	19	79.2	18	75.0	14	58.3	18	75.0
**Type 2**	74	73	98.6	71	95.9	74	100.0	62	83.8	73	98.6	60	81.1	68	91.9	62	83.8	70	94.6
**Type 3**	117	116	99.1	106	90.6	61	52.1	54	46.2	102	87.2	25	21.4	109	93.2	112	95.7	48	41.0
**Others**	12	12	100.0	11	91.7	11	91.7	9	75.0	11	91.7	9	75.0	12	100.0	11	91.7	9	75.0
**All**	227	224	98.6	210	92.5	169	74.4	136	59.9	207	91.2	113	49.8	207	91.2	199	87.7	145	63.9

^a^The number of EGF proteins of a specific type, ^b^A hit for a sub-domain occurred when more than half of the sub-domain residues were contained in a given motif. We present the number of hits of different types, ^c^Cov(Coverage) was defined as the ratio of the number of hits to the number of EGF proteins, e.g., if No. = 24 and Hits = 22, then Cov = 22/24 = 91.7%.

**Table 11:** Statistical analysis of EGF(EGF-like) proteins whose sub-domains were detected by MEME

	**Structural Alphabet**					
**EGF proteins**	**Our SA**		**Yang & Tung's**		**de Brevern *et al*.'s**	
	
	**Count**	**Percentage**	**Count**	**Percentage**	**Count**	**Percentage**

**Found 3**^**a**^	151	66.52	79	34.80	104	45.81
**Found 2**^**b**^	74	32.60	78	34.36	116	51.10
**Found 1**^**c**^	2	0.88	63	27.75	7	3.08
**Found 0**^**d**^	0	0.00	7	3.08	0	0.00
**Total**	227	100.00	227	100.00	227	100.00

^a^EGF (EGF-like) proteins in which all three sub-domains (A, B and C) were found by MEME, ^b^EGF (EGF-like) proteins in which two of the three sub-domains were found by MEME, ^c^EGF (EGF-like) proteins in which only one sub-domain was found by MEME, ^d^EGF (EGF-like) proteins in which MEME failed to identify any sub-domain.

## Discussion

This study aimed to: (1) introduce a systematic and modular pipeline for protein structural alphabet design, and (2) analyze the potential of our new alphabet to characterize local protein properties. There are two features that distinguish our method from the others. First, we took a multi-strategy approach to structural alphabet design. The alphabet size was automatically and statistically determined based on BIC and was visualized using a unified distance matrix (U-matrix). We did not pre-specify the alphabet size [24] or use an ad hoc procedure, such as iterative shrinking, to find the optimal size [15]. And, unlike other methods that use specialized databases, e.g. Pair Database [24] and PDB-SELECT [32,42], the protein structure data used to build the alphabet were obtained from the non-redundant PDB (nrPDB) database and were not pre-processed for any particular purpose, ensuring the generality of our alphabet. Second, we proposed a novel automatic matrix training framework to construct an appropriate substitution matrix for the alphabet. This training strategy did not need any information about known alignments, e.g. PALI [43], that most previous strategies have required. Using different training data and update rules, the self-training methodology can be applied to various alphabets. For example, instead of protein classifications, we could consider RMSD in the update rules to tune the matrix. In Table 12, we summarize the properties of the structural alphabets and design methods evaluated in this study.

**Table 12:** Summary of properties of structural alphabets and alphabet designs

**Structural Alphabet**	**Tung & Yang**	**de Brevern et al**.	**Our SA**
**Alphabet Size**	23	16	18
**How the alphabet size was determined**	Prespecified	Iterative shrinking	BIC
**Clustering**	k-means	SOM+HMM	SOM+k-means
**Data Set**	Preprocessed (Pair Database)	Preprocessed (PBE-SELECT)	No preprocess (nrPDB)
**Substitution Matrix**	BLOSUM-like	BLOSUM-like	Self-Training
**Requires known alignments to build matrix**	Yes	Yes	No
**Applicability**	Limited	Limited	Modular design More flexible

We demonstrated that our pipeline could produce a biologically meaningful structural alphabet. We compared SA-FAST, a search tool based on FASTA combined with our alphabet and substitution matrix, with other search tools. The results showed that SA-FAST was very competitive in its predictive accuracy and alignment efficiency for database-scale searches. In addition, we compared our alphabet with others in difficult cases of pairwise alignment. The number of residues aligned and the RMSD superpositions indicated that our structural alphabet was not only comparable to other alphabets but also performed competitively with structural alignment tools.

We found several advantages to using a 1D structural alphabet. First, 1D representations of protein structures are easier to compare and more economical to store. Second, previously designed and widely used 1D sequence alignment tools can be applied directly to protein structure and sequence analysis. Third, 1D-based approaches can serve as pre-processors to filter out irrelevant proteins prior to the application of more computationally intensive structural analysis tools.

## Conclusion

These results are encouraging and we can extend this work in several directions. Firstly, we can use more complete datasets for substitution matrix training to increase the sensitivity and selectivity of future database searches. Secondly, we can combine other alignment tools, in addition to FASTA, with our substitution matrix and evaluate the performance of these different combinations. Thirdly, to increase the performance of MEME in structural motif detection, we could modify MEME or develop a new motif-finding tool specifically for our structural alphabet. MEME was originally designed to find motifs in amino acid and nucleic acid sequences. Currently, we use MEME to detect protein motifs and we have demonstrated that it can recover some of the structural sub-domains described by our structural alphabet. Finally, several structural alphabets have been developed based on different protein structural characteristics. It would be worthwhile to conduct a thorough comparative study and evaluate the feasibility of combining different alphabets. The combination of complementary structural alphabets would increase their overall applicability and characterize 3D protein structures more completely.

## Methods

The use of frequent local structural motifs embedded in a polypeptide backbone has recently been shown to improve protein structure prediction [1,22]. The success of this strategy has paved the way for further studies of structural alphabets and has enabled the application of standard 1D sequence alignment methods to 3D protein structural searches. In this study, we combined several computational methods into a new approach to the design of a protein structural alphabet. We then developed an automatic matrix training framework that could generate appropriate substitution matrices for new alphabets when applied in standard 1D sequence alignment methods, such as FASTA [27].

### Structural alphabet design

We used proteins from the nrPDB [44] in our study with the aim of building a structural alphabet suitable for all proteins. The same approach could easily be applied to other databanks as well. We transformed each protein backbone into a series of dihedral angles (*ϕ *and *ψ*, neglecting *ω*) [15,22]. Following de Brevern *et al*. [15], our analysis was limited to fragments of five residues because this number of residues is sufficient for describing a short *α *helix and a minimal *β *structure. Fixing the window size at five residues, we applied a sliding-window approach to each protein sequence in nrPDB and gathered 20,953,584 fragment vectors. Each protein fragment, associated with *α*-carbons *C*_*α*(*i*-2)_, *C*_*α*(*i*-1)_, *C*_*α*(*i*)_, *C*_*α*(*i*+1)_, and *C*_*α*(*i*+2)_, was represented by a vector of eight dihedral angles [*ψ*_*i*-2_, *ϕ*_*i*-1_, *ψ*_*i*-1_, *ϕ*_*i*_, *ψ*_*i*_, *ϕ*_*i*+1_, *ψ*_*i*+1_, *ϕ*_*i*+2_] Unlike previous works that directly applied SOMs to obtain clusters of backbone fragments as the basis of the structural alphabet [28], in our approach the SOM was only part of the process that determined the number of letters required for the alphabet. We did not build our alphabet directly from the clusters found by SOM.

The U-matrix is one of the most widely used methods for visualizing the clustering results of the SOM. The U-matrix shows the distances between neighboring reference vectors and can be visualized efficiently using the greyscale [45]. We conducted a post-process on the U-matrix using a minimum spanning tree algorithm. Based on the grey levels in the U-matrix, all of the map units were linked in the minimum spanning tree. Given a threshold *θ *determined by BIC, we partitioned the entire tree into several disconnected subtrees by removing the links between map units with grey levels below *θ*.

Let *S *= {*s*_*i *_| *i *= 1...*M*} be the set of map units we wished to cluster. Each map unit *s*_*i *_is associated with a collection of input data points, $X^{i}=\{x_{j}^{i}|j=1...n_{i}\}$, mapped to the map unit *s*_*i*_. Let *C*_*k *_= {*c*_*i *_| *i *= 1...*k*} be the clustering of map units *S *with *k *clusters. We modeled each cluster *c*_*i *_as a multivariate Gaussian distribution *N*(*μ*_*i*_, *Σ*_*i*_), where *μ*_*i *_and *Σ*_*i *_were estimated as the sample mean and the sample covariance from *X*^*i*^, respectively. The number of parameters for each cluster was thus $d+\frac{1}{2}d(d+1)$, where *d *= 8 in our case. We defined *BIC*(*C*_*k*_) as:

$$BIC(C_{k})=\sum_{i=1}^{k}\{-\frac{1}{2}n_{i}\log\left|\sum_{i}\right|\}-\lambda \frac{1}{2}(d+\frac{1}{2}d(d+1))k\log N$$

where $N=\sum_{i=1}^{k}n_{i}$ and *λ*, the penalty weight, was set to 1.

We chose the threshold *θ *that maximized *BIC*(*C*_*k*_). For example, for an SOM with 200 × 200 map units, the threshold *θ *that maximized *BIC*(*C*_*k*_) was 21. The number of subtrees becomes the structural alphabet size. Because the SOM can be viewed as a topology preserving mapping from input space onto the 2D grid of map units, the number of map units can affect the clustering result. We systematically varied the number of units and repeated the above process. We selected the most frequent number of clusters as the alphabet size. After a series of systematic tests, we found that 18 was the most frequent number of clusters; therefore, 18 letters became size of our structural alphabet.

Rather than adopt the two-level approach that first trains the SOM then performs clustering on the trained SOM after determining the alphabet size [28], we applied the k-means algorithm to the input data vectors directly to obtain the clusters. The SOM established a local order among the set of reference vectors such that the closeness between two reference vectors in the *R*^*d *^space was dependent on how close the corresponding map units were in the 2D array. Nevertheless, an inductive bias of this kind might not be appropriate for structural alphabets since the local order does not always faithfully characterize the relationship between structural building blocks and can sometimes be misleading. For example, forcing the topology to preserve mapping from the input space of *α*-helix and *β*-strand to a 2D grid of units could be harmful to clustering. Therefore, we used the SOM only to visualize the alphabet size and relied on the k-means algorithm to extract the local features directly from the input data that actually reflected the characteristics of the clusters. The centroid of each cluster forms the prototypical representation of each alphabet letter. We performed k-means clustering 50 times, starting with different random seeds, all using k = 18. We computed the Euclidean distances from each fragment in each cluster to its centroid as the intra-cluster distance; we also calculated the centroid-to-centroid distance. We kept the clustering result that had the minimum ratio of the average intra-cluster distance to the centroid-to-centroid distance. Given this result as the basis for the structural alphabet, we could transform a protein into a series of alphabet letters by matching each of its fragments against our alphabet prototypes.

### Automatic substitution matrix training

The substitution matrix used to align proteins represented by structural alphabets affects the alignment accuracy. The matrix is a crucial factor in the success of applying a 1D sequence alignment tool to search for similar 3D structures. The simplest matrix that can be used is the identity matrix. Some authors have applied an HMM approach to define the matrix [33], while others have adopted approaches similar to the development of BLOSUM matrices [24,31,45]. The identity matrix ignores possible acceptable alphabet letter substitutions, significantly limiting its applicability. The BLOSUM-like approach requires alignments of homologous proteins before calculating the log-odd ratios as the entries in the matrix; however, reliably aligned protein blocks might not always be available for log-odd ratio estimation. To avoid these drawbacks, we trained the substitution matrix without using the known blocks of protein alignments. This matrix training procedure can be applied regardless of how the alphabet is derived.

There are three components in the matrix training framework: an alignment tool with a substitution matrix, training data, and a matrix trainer. We used FASTA as the alignment tool and the non-redundant proteins in SCOP1.69 with sequence similarity less than 40%, excluding the families with less than five proteins and those proteins used for validation, as the training dataset. Note that the training dataset was only 9.62% of the entire SCOP1.69. The test data we used in the later experiments (see Results section) did not overlap with our training examples. We started by using the identity matrix as the initial substitution matrix where the score for a match was 1, and for a mismatch, 0. Each protein in the training dataset was iterated as a query for FASTA to search the rest of the dataset for similar proteins. If a protein returned by FASTA belonged to the same family as the query, we considered the case a positive hit; otherwise it was a negative hit. Those proteins not returned by FASTA but in the same family as the query were considered misses. We gathered the alignments of all positive hits and misses and computed the log-odd ratios to build the *positive matrix *based on the alignments. Similarly, we constructed the *negative matrix *using the alignments of negative hits, The matrix trainer updated the current substitution matrix *S*^(*t*) ^to *S*^(*t*+1) ^as follows:

*S*^(*t*+1) ^= *S*^(*t*) ^+ *M*

*M *= [*W*_*p*_·(*P *- *S*^(*t*)^) - *W*_*n*_·(*N *- *S*^(*t*)^)]·*τ*

*W*_*p *_= (|*positive_hits*| + |*misses*|)/|*taining_data*|

**Figure 7:**
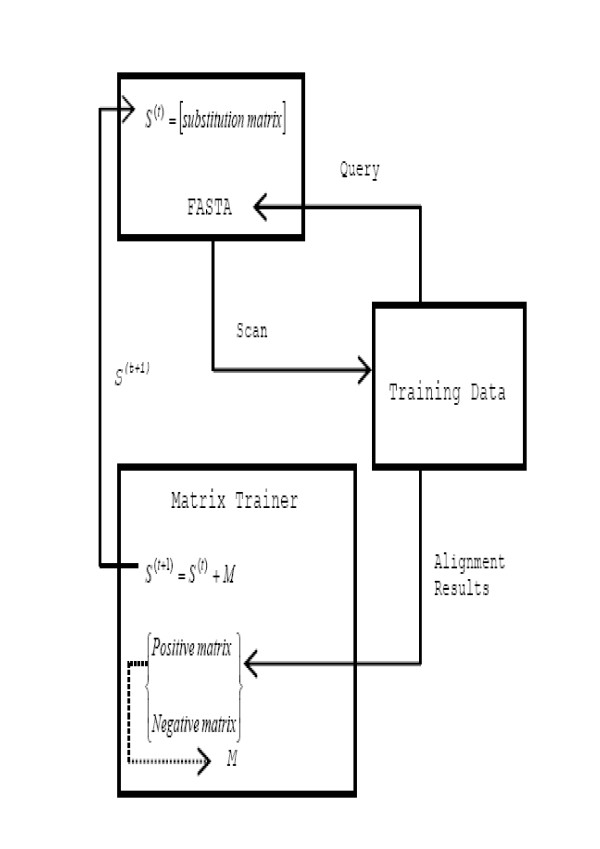
System architecture of the matrix training framework.

*W*_*n *_= |*negative_hits*|/|*training_data*|

where *P *and *N *are the positive and the negative matrix, respectively, *τ *is the learning rate (similar to the learning rate in neural networks), and *W*_*p *_and *W*_*n *_are the weights. The weights were defined as the proportion of the total number of positive hits and misses to the training data size and the ratio of the number of negative hits to the training data size, respectively. We repeated the update process to train the substitution matrix until there were no changes in the matrix, that is, the number of both the positive and the negative hits remained constant. This converged matrix was the final substitution matrix that we combined with FASTA to become a new alignment tool named SA-FAST. SA-FAST was used to demonstrate the applicability of our new alphabet and matrix. The training framework appears in Figure 7.

## Authors' contributions

S–YK implemented the structural alphabet design pipeline and conducted the experiments. Y–JH designed the BIC procedure, the matrix training framework and experiments, and supervised this study. Both authors read and approved the final manuscript.
